# Voice-based prediction of prediabetes using classical machine learning models

**DOI:** 10.3389/fcdhc.2025.1697769

**Published:** 2025-11-27

**Authors:** Jessica Oreskovic, Ghazal Fazli, Vanita Varma, Kinza Malik, Jaycee Kaufman, Yan Fossat

**Affiliations:** 1 Klick Applied Sciences, Klick, Inc., Toronto, ON, Canada; 2 Department of Geography, Geomatics and the Environment, University of Toronto Mississauga, Mississauga, ON, Canada; 3 Center for Innovation in Health and Wellness, Humber Polytechnic, Toronto, ON, Canada; 4 Faculty of Health &Life Sciences, Humber Polytechnic, Toronto, ON, Canada

**Keywords:** prediabetes, voice, vocal biomarker, type 2 diabetes, voice signal analysis

## Abstract

**Introduction:**

Prediabetes is a highly prevalent metabolic condition that significantly increases the risk of developing type 2 diabetes and cardiovascular disease. Despite its clinical importance, over 80% of individuals with prediabetes remain undiagnosed. Voice analysis has emerged as a non-invasive, accessible method for disease screening, with prior work showing promising results in detecting hypertension and type 2 diabetes from acoustic features. This study investigates whether voice-based machine learning models can identify individuals with prediabetes and evaluates the generalizability of these models across populations.

**Methods:**

Participants were recruited from clinical sites in India and a community college in Canada. All participants recorded the same spoken phrase multiple times daily via a mobile app, and glycemic status was assessed using HbA1c levels. Voice recordings were preprocessed to remove silence and trimmed to exclude potentially uninformative sections. A total of 167 acoustic features were extracted from each sample using Librosa, scipy, and parselmouth. Features were averaged per participant. Sex-specific models were developed under six experimental configurations varying by dataset balance (age/BMI-matched vs. unbalanced) and BMI inclusion. Feature selection was conducted using L1-regularized logistic regression (LASSO), and SMOTE was applied during training to address class imbalance. Twelve machine learning classifiers were evaluated using leave-one-subject-out cross-validation (LOSO-CV) on the India dataset. Final models were tested on a holdout India subset and the independent Canada dataset.

**Results:**

In cross-validation, the best female model (XGBoost, balanced, no BMI) achieved a balanced accuracy of 0.78, and the best male model (Random Forest, balanced, no BMI) achieved 0.68. However, holdout set testing identified different optimal configurations for generalization: the male XGBoost model trained on an unbalanced dataset outperformed the cross-validated model. In the Canada dataset, models failed to generalize effectively, with several configurations unable to correctly identify prediabetic participants.

**Discussion:**

Voice-based prediction models show potential for prediabetes screening in controlled populations, but their performance declines when applied across geographic or demographic boundaries. These findings highlight the need for more diverse training data and population-specific model tuning to support real-world applicability.

## 1 Introduction

Prediabetes is a chronic metabolic condition characterized by blood glucose levels that are higher than normal but not yet meeting the diagnostic criteria for type 2 diabetes (fasting plasma glucose 100–125 mg/dL or HbA1c 5.7–6.4%) (1). Globally, the age-adjusted prevalence of impaired glucose tolerance is estimated at 9.1%, translating to hundreds of millions of adults at risk for progression to overt diabetes (2). In the United States alone, approximately 98 million adults have prediabetes, yet over 80 percent are unaware of their condition 3). Individuals with prediabetes face an increased risk of progression to type 2 diabetes as well as elevated risk of cardiovascular disease and stroke even before overt hyperglycemia develops (3). The economic burden of prediabetes is substantial with direct and indirect costs associated with prediabetes in the U.S. were estimated at $43.4 billion in 2017 (4). Lifestyle interventions such as modest weight loss through healthy diet and increased physical activity can reduce the risk of progression by up to 50 percent (3, 5), underscoring the need for proactive screening and early intervention to mitigate individual and population-level impacts.

The prevalence of prediabetes has considerable regional variation. Although North America reports age-adjusted impaired glucose tolerance and impaired fasting glucose prevalences of 13.1% and 7.6%, respectively (2, 6, 7), recent evidence from South Asia indicates an even higher burden. A 2025 meta-analysis encompassing over 150,000 adults across eight South Asian countries found a pooled prediabetes prevalence of 18.99% (95% CI 12.74–26.6), nearly twice the global average (8). Given the interaction of genetic, environmental, and lifestyle factors unique to South Asian populations, there is a clear rationale for developing and validating screening strategies that reflect their specific demographic and lifestyle characteristics to enhance early detection and inform targeted prevention efforts.

Early metabolic changes characteristic of prediabetes such as mild hyperglycemia, low-grade inflammation, autonomic dysregulation, and incipient neuropathy can plausibly affect vocal fold tissue viscosity and neuromuscular control, producing subtle shifts in phonatory stability (e.g., F0, jitter/shimmer, HNR) and intensity regulation (9–11). Recent work shows that voice features can vary with glucose levels in type 2 diabetes and distinguish overt metabolic and cardiovascular conditions such as T2D and hypertension, suggesting that related but smaller effects may exist at earlier stages in disease progression (12–14). A voice-based screening method is also non-invasive, accessible, low-cost, and easily repeatable at scale, making it a practical front-end to guide confirmatory HbA1c testing particularly in settings with limited access to laboratory screening. On this basis, we hypothesized that multivariate acoustic patterns would capture a subtle, distributed signal of prediabetes, and we evaluated this using sex-specific models trained on a fixed sentence to maximize standardization across cohorts.

In this paper, we cover the development of a machine-learning model that leverages acoustic features of a fixed sentence to identify healthy or prediabetic individuals. We evaluate the model's performance in an independent validation cohort from a demographically and geographically distinct population. By deliberately assessing performance across geographically diverse groups, we aim to map the practical limits of acoustic-feature discrimination of prediabetes. This cross-population evaluation will uncover how factors such as age, dialect, and recording environment influence model robustness. In doing so, we establish a benchmark for the upper and lower bounds of voice-based screening performance, offering a clear framework for future work to expand dataset diversity, refine feature extraction, and bolster generalizability before clinical deployment.

## 2 Methods

### 2.1 Participant recruitment

This study used primary data collected under IRB-approved protocols in India and Canada. Participants were recruited in two parts: first, within a study conducted in India and second, within a study conducted in Canada.

In the first study, referred to as the "India Dataset", participants were recruited from three sites: Datta Meghe Medical College, Mavens Hospital, Mittal Global Clinical Trial Services. Participants were recruited as part of a larger study analyzing vocal acoustic changes with changing blood glucose (Clinical Trial Identifier: CTRI/2021/08/035957). Participants were clinically assessed and categorized as nondiabetic, prediabetic, or type 2 diabetic based on diagnostic guidelines from the American Diabetes Association. For this analysis, only individuals classified as nondiabetic or prediabetic were included; those with type 2 diabetes were excluded. Participant age, BMI, heart rate, blood pressure, and HbA1c were measured and recorded at recruitment. We excluded smokers and individuals with, acute illnesses (e.g., upper respiratory infections) or chronic medical conditions other than T2D to minimize acoustic confounding variables unrelated to glycemic status. All participants were fluent in English and signed informed consent. The study protocol was approved by three ethics committees (Jasleen Hospitals Ethics Committee, Mavens Institutional Ethics Committee & Saanvi Ethical Research LLP). All methods were conducted in accordance with relevant guidelines and regulations.

In the second study, referred to as the "Canada Dataset", participants who identified as South Asian were recruited from Humber Polytechnic, a public college located in Toronto, Canada. It should be noted that this cohort is not representative of the broader Canadian population. Participant age, biological sex, gender, and BMI were self-reported through a survey. HbA1c testing was conducted using the Abbott Afinion analyzer (Abbott, USA) by trained staff. All participants were fluent in English and had no known diagnosis of acute illnesses (e.g., upper respiratory infections) or chronic medical conditions. All participants signed informed consent. The study protocol was approved by the Humber Polytechnic Research Ethics Board. All methods were conducted in accordance with relevant guidelines and regulations.

In both studies, participants downloaded a custom mobile application for data collection onto their personal smartphones. This application permits easy collection of de-identified voice recordings for use in research studies. Upon downloading the application, participants log in with an anonymized alpha-numeric participant identifier. When they record a voice sample, the sample is automatically uploaded to a secure, private Firebase server (Google, USA), and the voice sample is removed from the personal device. No personal information was collected in the voice recordings, and participants in both studies recorded the voice segment "Hello, how are you? What is my glucose level right now?" in a normal tone and were asked to deliver the phrase consistently throughout the study. In the India Dataset, participants were instructed to record their voice up to six times daily for two weeks, and in the Canada Dataset, participants were instructed to record their voice at least three times daily for five days. The reduction in required number of recordings was implemented to decrease subject burden and was informed by analysis on the India dataset which found that fewer recordings could achieve similar results (15).

### 2.2 Audio processing and quality assurance

The Speech Recognition Python library was employed to transcribe each audio recording, and excess silence at the beginning and end of the audio segments were removed using a voice activity detector (webrtcvad Python package, which wraps the WebRTC VAD developed by Google, aggressiveness set to 3). Only recordings containing the stipulated phrases ("Hello, how are you? What is my glucose level right now?") were included in the analysis.

### 2.3 Voice feature extraction

In total, 167 voice features were extracted from each recording. To improve feature quality, the first and last quarters of each recording were removed prior to extraction. This decision was informed by prior work showing that the first quarter contributed minimally to model-relevant features, and the final quarter was excluded to reduce the risk of capturing trailing silence or background noise (14). Acoustic features were extracted from raw voice recordings using a custom Python pipeline incorporating Librosa (0.11.0), scipy (1.12.0), and parselmouth (0.4.6) (16). This included both standard speech features (MFCCs, spectral and temporal statistics) and phonation-based measures (pitch, jitter, shimmer,etc) derived from Praat functions. Entropy-based and bandpower features were also computed to capture signal complexity and energy distribution. The feature set aimed to comprehensively represent vocal characteristics potentially affected by metabolic changes.

We formed a subject-level representation by averaging all 167 acoustic features across each participant's recordings to reduce transient within-subject noise and align with the trait-level endpoint (normoglycemic vs. prediabetic). Feature dimensionality was reduced before model development. To assess within-participant variability, we ran an exploratory one-way random-effects variance decomposition on per-utterance data, estimating pooled within-subject and adjusted between-subject components (accounting for unequal repeats) and reporting per-feature ICCs; features with insufficient repeats were excluded from within-subject estimates. This analysis was descriptive and did not inform training or tuning.

### 2.4 Vocal parameter statistical analysis

Acoustic parameters were analyzed for statistical significance between normoglycemic and prediabetic participants using Mann Whitney U test for significance. In total, seven tests were performed for each sex, corresponding to the pitch, standard deviation of the pitch, intensity, standard deviation of the intensity, harmonic noise ratio, shimmer, and jitter for each participant. To address multiple comparisons, we controlled the false discovery rate using the Benjamini–Hochberg procedure (BH–FDR; q=0.05) as well as applied Bonferroni family-wise error-rate control as a sensitivity analysis (α=0.05; Bonferroni-adjusted p-values also reported).

### 2.5 Voice prediction models

Overall, model development was conducted similarly to prior work (14).

Model development was done using the India dataset and only subjects who were diagnosed normoglycemic or prediabetic according to ADA guidelines (prediabetic HbA1c 5.70-6.49%). Participants in the type 2 diabetes range were excluded from this analysis.

Given the known physiological and acoustic differences in voice characteristics between males and females as well as sex-specific patterns in diabetes pathophysiology, we developed separate models for male and female participants (17). To mitigate class imbalance between prediabetic and normoglycemic individuals, we constructed sex-specific balanced training sets using stratified subsampling in age–BMI space. Specifically, we retained all prediabetic participants (cases) and discretized age and BMI into five equal-width bins computed from the full training set; within each age×BMI cell, we randomly sampled normoglycemic controls in proportion to the number of cases in that cell (up to a 3:1 control:case ratio), using a fixed random seed. This procedure preserves the joint distribution of age and BMI while maximizing sample size. To assess the impact of class imbalance and anthropometric normalization, models were developed and evaluated on both the age/BMI balanced and original (unbalanced) datasets.

To further examine the contribution of BMI as a predictive feature, we performed an additional set of analyses with BMI included as an input variable for the unbalanced dataset. This resulted in six experimental configurations: age/BMI balanced or unbalanced with and without BMI as a feature, for male and female subgroups respectively.

Starting with 167 features, feature reduction was performed to reduce dimensionality and prevent overfitting using L1-regularized logistic regression (LASSO). This method reduces feature dimensionality by penalizing less informative variables, resulting in a more concise and predictive feature set. A range of regularization strengths (alpha values) were systematically tested, and performance was evaluated using leave-one-subject-out cross-validation (LOSO-CV) to approximate real-world, subject-independent performance. The optimal alpha was selected based on the configuration that maximized average classification accuracy across LOSO folds. The resulting reduced feature sets varied by sex/configuration and contained ~18–33 features, and downstream classifiers were trained on these subsets.

To address class imbalance during training, the Synthetic Minority Oversampling Technique (SMOTE) was applied to the minority class within each LOSO-CV training fold. SMOTE synthetically generates new samples by interpolating between existing minority class instances and their nearest neighbors (18). This approach has been previously described in detail in our prior work (14), where it was shown to improve classifier generalizability. As in that study, SMOTE was applied only to the training data within each fold to avoid data leakage.

For model development, we evaluated 12 classical machine learning algorithms: Support Vector Classifier (SVC), Nu-Support Vector Classifier (NuSVC), K-Nearest Neighbors (KNN), Logistic Regression (LR), Linear Discriminant Analysis (LDA), Quadratic Discriminant Analysis (QDA), Gaussian Naïve Bayes (GNB), Bernoulli Naïve Bayes (BNB), Ridge Classifier, eXtreme Gradient Boosting (XGBoost), Decision Tree (DT), and Random Forest (RF). All models were implemented using the scikit-learn implementations with default hyperparameters under the same preprocessing and stratified cross-validation. This design provides a fair, reproducible baseline across diverse inductive biases while minimizing overfitting risk in a small-N setting.

Model discrimination was assessed using the area under the receiver operating characteristic curve (AUC) with 95% confidence intervals obtained by stratified bootstrap resampling on out-of-sample predictions from the evaluation procedure. Calibration was summarized using the Brier score (mean squared error between predicted probabilities and observed outcomes) alongside balanced accuracy, sensitivity, specificity, and F1. All evaluations used left-out predictions from the LOSO procedure without refitting on test instances.

After training, the best-performing models from each configuration were evaluated using a holdout test set consisting of a subset of unseen subjects drawn from the India study. The holdout set included 26 females (7 prediabetic), and 45 males (12 prediabetic). Finally, the Canada dataset was also tested on these models to study the extendibility of the models and assess their performance on alternate populations. This final validation step allowed us to assess generalizability across populations.

## 3 Results

### 3.1 Participant demographic information

After quality control of the voice recordings, each participant recorded an average of 31.44 +/- 19.68 recordings in the India dataset and 15.24 +/- 13.62 recordings in the Canada dataset. The large variation in the Canada dataset can be attributed to five participants recording between 40–80 voice samples and one participant recording over 100 voice samples. In total, 11235 recordings were collected in the India dataset, and 1997 recordings were collected in the Canada dataset.

Overall, 128 females (95 normoglycemic and 33 prediabetic) and 231 males (171 normoglycemic and 60 prediabetic) were recruited in the India study. In the Canada study, 90 females (83 normoglycemic and 7 prediabetic) and 41 males (36 normoglycemic and 5 prediabetic) were recruited. Age, BMI, and HbA1c were collected at recruitment in the India Dataset and are displayed in **Table 1** along with the age ranges, BMI, and HbA1c that were collected at recruitment in the Canada Dataset.

**Table 1:** Participant demographic information for india dataset at recruitment.

		Female		Male	
		Normoglycemic	Prediabetic	Normoglycemic	Prediabetic
	N	95	33	171	60
India dataset	Age (years)	34.24 +/- 11.72	44.77 +/- 12.62	34.67 +/- 12.14	44.37 +/- 12.50
	BMI (kg/m^2)	27.23 +/- 5.8	29.65 +/- 5.48	26.95 +/- 4.44	27.39 +/- 3.89
	HbA1c (%)	5.08 +/- 0.36	6.06 +/- 0.21	5.11 +/- 0.38	6.07 +/- 0.21
	N	83	7	36	5
Canada dataset	Age 18 to 23 24 to 29 30 to 34	63 16 4	6 1 0	27 8 1	5 0 0
	BMI (kg/m^2)	22.16 +/- 3.91	23.9 +/- 2.25	22.7 +/- 3.49	23.49 +/- 5.08
	HbA1c (%)	5.28 +/- 0.20	5.94 +/- 0.28	5.31 +/- 0.21	5.76 +/- 0.09

### 3.2 Acoustic variables

A subset of seven handcrafted acoustic variables (F0,F0-SD, Intensity, Intensity SD, HNR, Shimmer, and Jitter) were analyzed between normoglycemic and prediabetic populations because they are clinically interpretable and widely reported in voice analysis. Although some features displayed a shift between diagnostic classes, such as a decrease in F0 in prediabetic females, there were no statistically significant differences in acoustic features between the India Dataset and the Canada Dataset (**Tables 2**, **3**). Across both cohorts and sexes, none of the seven prespecified acoustic features differed significantly after Bonferroni family-wise error control (α=0.05) or Benjamini–Hochberg false discovery rate adjustment (q<0.05) (**Tables 2**, **3**), indicating that any class-related signal is subtle and likely multivariate in nature.

**Table 2:** Acoustic variables obtained from participant voice recordings in the India dataset.

Features	Female – India dataset				Male – India dataset			
	Normoglycemic (N = 95)	Prediabetic (N = 33)	P-value (Bonferroni)	P-value (BH-FDR)	Normoglycemic (N = 171)	Prediabetic (N = 60)	P-value (Bonferroni)	P-value (BH-FDR)
F0 (Hz)	217.47 [203.44-232.79]	203.19 [180.98-225.46]	0.19	0.19	140.64 [126.5-158.07]	139.0 [127.0-163.0]	>0.99	0.96
F0SD (Hz)	36.47 [30.0-43.38]	34.81 [27.01-39.97]	>0.99	0.81	25.23 [19.19-33.28]	23.26 [18.25-33.90]	>0.99	0.71
Intensity (dB)	62.81 [58.06-65.33]	61.73 [56.21-65.46]	>0.99	0.81	64.82 [59.55-68.08]	62.86 [59.44-67.07]	>0.99	0.49
IntensitySD (dB)	11.03 [9.06-13.8]	10.21 [8.67-13.52]	>0.99	0.93	11.03 [9.05-14.29]	12.82 [9.52-16.38]	0.74	0.36
HNR	12.85 [11.6-13.92]	13.09 [11.76-13.69]	>0.99	0.93	10.93 [9.64-12.4]	11.45 [9.77-13.04]	>0.99	0.49
Shimmer	0.11 [0.10-0.12]	0.11 [0.10-0.12]	>0.99	0.93	0.11 [0.10-0.13]	0.11 [0.10-0.13]	>0.99	0.86
Jitter	0.019 [0.018-0.022]	0.020 [0.018-0.021]	>0.99	0.93	0.021 [0.019-0.023]	0.020 [0.018-0.023]	>0.99	0.71

Values are presented as median [first quartile - third quartile]. p-values were calculated with two-sided Mann–Whitney U tests and adjusted for multiple comparisons using the Bonferroni method (FWER, α=0.05), as well as the Benjamini–Hochberg false discovery rate (FDR) procedure (q-values; q<0.05 considered significant). F0, Fundamental frequency; SD, standard deviation; HNR, harmonic noise ratio.

**Table 3:** Acoustic variables obtained from participant voice recordings in the Canada dataset.

Features	Female – Canada dataset				Male – Canada dataset			
	Normoglycemic (N = 83)	Prediabetic (N = 7)	P-value (Bonferroni)	P-value (BH-FDR)	Normoglycemic (N = 36)	Prediabetic (N = 5)	P-value (Bonferroni)	P-value (BH-FDR)
F0 (Hz)	221.94 [204.29-235.14]	219.4 [215.61-238.7]	>0.99	0.73	125.75 [116.98-141.88]	136.22 [107.2-147.8]	>0.99	0.83
F0SD (Hz)	35.06 [30.25-38.72]	31.59 [26.57-37.74]	>0.99	0.73	17.33 [13.55-21.73]	24.61 [18.65-28.86]]	>0.99	0.53
Intensity (dB)	60.53 [56.29-65.34]	62.5 [59.81-63.73]	>0.99	0.73	57.42 [54.01-60.86]	58.18 [56.88-62.69]	>0.99	0.83
IntensitySD (dB)	13.71 [11.56-18.34]	13.91 [10.95-21.21]	>0.99	0.88	13.85 [12.33-20.26]	15.78 [15.34-20.12]	>0.99	0.53
HNR	16.61 [14.59-18.08]	16.74 [15.44-17.96]	>0.99	0.73	12.51 [10.37-13.62]	14.4 [12.34-14.67]	>0.99	0.53
Shimmer	0.09 [0.08-0.1]	0.09 [0.07-0.09]	>0.99	0.73	0.11 [0.10-0.12]	0.11 [0.10-0.12]	>0.99	0.83
Jitter	0.017 [0.015-0.020]	0.016 [0.014-0.016]	0.49	0.49	0.022 [0.020-0.026]	0.018 [0.017-0.025]	>0.99	0.53

Values are presented as median [first quartile - third quartile]. p-values were calculated with two-sided Mann–Whitney U tests and adjusted for multiple comparisons using the Bonferroni method (FWER, α=0.05), as well as the Benjamini–Hochberg false discovery rate (FDR) procedure (q-values; q<0.05 considered significant). F0, Fundamental frequency; SD, standard deviation; HNR, harmonic noise ratio.

**Figure 1:**
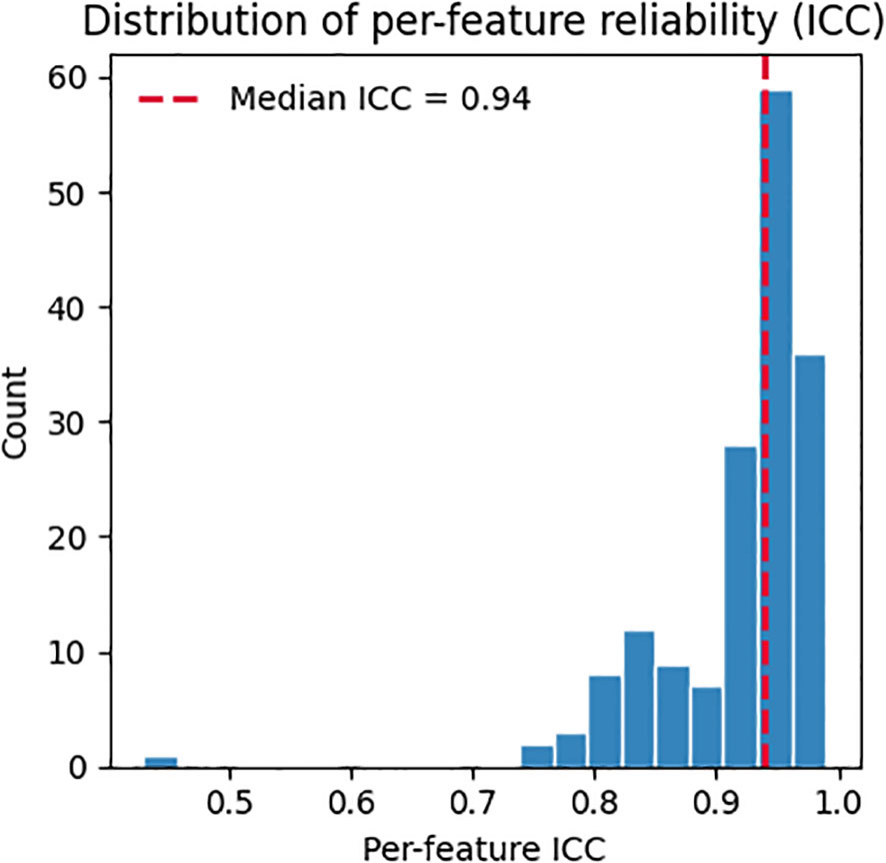
Per-feature reliability (ICC) estimated via random-effects variance decomposition. Vertical dashed line marks the median across features.

Across 571 participants (median 28 utterances/participant), adjusted between-subject variability exceeded within-subject variability on average (mean pooled within-SD 95.0, mean adjusted between-SD 285.5). Many features showed high reliability (ICC ≳ 0.95) (**Figure 1**), including meanF0Hz (ICC 0.9895) and spectral measures such as spec_rolloff_mean (ICC 0.9733), spec_centroid_mean (ICC 0.9689), and band-power features (e.g., 30–40 Hz; ICC 0.9744). These results indicate the signal is largely stable within a participant, with smaller day-to-day fluctuations relative to between-participant differences.

### 3.3 Voice prediction models

Of the experimental configurations, the model developed using the age/BMI balanced dataset performed best for both males and females in the cross-validation (**Table 4**). For males, the model with the best balanced accuracy for the leave-one-subject-out cross validation was a random forest classifier with a reduced feature set of 33 features. The balanced accuracy was 0.68, sensitivity of 0.55, specificity of 0.81, and F1 score of 0.60. For females, the best performing model on the cross validation was extreme gradient boosting classifier with a reduced feature set of 24 features. The model had a balanced accuracy of 0.78, sensitivity of 0.76, specificity of 0.81, and F1 score of 0.76. Cross-validated performance was modest (female best balanced accuracy 0.78; male best 0.68), and configurations selected by cross-validation did not always generalize to the independent India holdout (**Table 5**). We therefore report both CV and holdout metrics and interpret them cautiously.

**Table 4:** Model performance for each experimental configuration for leave-one-subject-out cross-validation.

Sex	Dataset	BMI feature	N_feats_	Model	Bacc	Sens	Spec	F1	AUC (95% CI)	Brier
Male	Unbalanced	Yes	18	BNB	0.66	0.66	0.66	0.51	0.65 (0.56-0.73)	0.26
		Feature set:		mfcc_mean10, mfcc_mean0, dmfcc_std1, bmi, bandPow0_10, mfcc_std0, mfcc_std10, mfcc_mean1, mfcc_std4, logEnt, mfcc_std8, kurt, meanF0Hz, mfcc_std12, spec_rolloff_mean, fundamental_freq, ddmfcc_std0, spec_centroid_std						
Male	Unbalanced	No	24	XGB	0.66	0.51	0.81	0.51	0.66 (0.58-0.75)	0.21
		Feature set:		mfcc_mean10, mfcc_mean12, tempgram_kurt, mfcc_std10, mfcc_std5, mfcc_std12, mfcc_std1, logEnt, ddmfcc_std6, bandPow0_10, spec_centroid_mean, spec_contrast_mean5, mfcc_mean9, dmfcc_std8, mfcc_std11, spec_contrast_std0, poly_std3, fundamental_freq, dmfcc_std0, spec_contrast_std6, dmfcc_std4, PXX_skew, ddmfcc_std1, spec_centroid_std						
Male	**Balanced**	**No**	**33**	**RF**	**0.68**	**0.55**	**0.81**	**0.60**	**0.68 (0.58-0.78)**	**0.22**
		Feature set:		spec_contrast_mean2, mfcc_mean4, diff_SMA, mfcc_mean12, mfcc_std1, dmfcc_std9, ddmfcc_std11, mfcc_mean10, spec_contrast_mean6, ddmfcc_mean2, spec_contrast_std6, ddmfcc_std2, mfcc_std11, mfcc_mean0, mfcc_std12, ddmfcc_std1, dmfcc_mean0, mfcc_mean9, mfcc_std10, dmfcc_std0, mfcc_mean8, skew, dmfcc_std3, mfcc_std7, fundamental_freq, spec_rolloff_mean, ddmfcc_std6, poly_std3, tempgram_kurt, dmfcc_std5, kurt, dmfcc_std4, spec_centroid_std						
Female	Unbalanced	Yes	26	LDA	0.69	0.60	0.77	0.53	0.68 (0.55-0.81)	0.23
		Feature set:		mfcc_std2, mfcc_mean7, dmfcc_std2, bandPow30_40, mfcc_mean0, bandPow0_10, stdevF0Hz, fundamental_freq, mfcc_mean6, mfcc_std5, mfcc_mean8, poly_std3, spec_contrast_mean1, ddmfcc_std6, ddmfcc_std5, HNR, spec_bandwith_std, meanF0Hz, ddmfcc_std1, bmi, PXX_logEnt, spec_bandwith_mean, ddmfcc_std3, logEnt, spec_contrast_std0, ddmfcc_std0						
Female	Unbalanced	No	23	DT	0.69	0.56	0.83	0.54	0.69 (0.59-0.80)	0.24
		Feature set:		dmfcc_std2, mfcc_std7, bandPow30_40, mfcc_mean3, spec_contrast_std6, stdevF0Hz, mfcc_mean11, mfcc_mean7, fundamental_freq, mfcc_mean0, mfcc_std6, mfcc_mean4, mfcc_std9, dmfcc_std0, mfcc_mean9, mfcc_mean12, dmfcc_std4, spec_bandwith_mean, meanF0Hz, spec_bandwith_std, tempgram_kurt, dmfcc_std1, spec_centroid_std						
Female	**Balanced**	**No**	**24**	**XGB**	**0.78**	**0.76**	**0.81**	**0.76**	**0.81 (0.69-0.92)**	**0.18**
		Feature set:		stdevF0Hz, ddmfcc_std10, spec_contrast_mean5, ddmfcc_std2, bandPow0_10, mfcc_mean4, stdevInten, meanF0Hz, dmfcc_std6, mfcc_mean5, spec_contrast_mean0, ddmfcc_std1, fundamental_freq, poly_mean3, mfcc_std11, spec_rolloff_mean, dmfcc_std3, logEnt, spec_bandwith_std, dmfcc_std0, diff_SMA, dmfcc_std7, tempgram_kurt, spec_centroid_std						

BNB, Bernoulli Naïve Bayes; XGB, eXtreme Gradient Boosting; RF, Random Forest Classifier; LDA, Linear Discriminant Analysis; DT, Decision Tree Classifier. Discrimination and calibration metrics per configuration. Values are computed on out-of-sample predictions. AUC (95% CI) from stratified bootstrap resampling; Brier is the mean squared error of predicted probabilities (lower is better).

**Table 5:** Experimental configurations model testing on India holdout set and Canada set.

	India holdout set				Canada set			
	Precision	Recall	F1-score	Balanced accuracy	Precision	Recall	F1-score	Balanced accuracy
#1 Male, Unbalanced, BMI included, BNB, 18 features								
Normoglycemic	0.70	0.48	0.57	–	1.00	0.09	0.16	–
Prediabetic	0.23	0.42	0.29	–	0.14	1.00	0.24	–
Overall	–	–	–	0.45	–	–	–	0.545
#2 Male, Unbalanced, XGBoost, 24 features								
Normoglycemic	0.81	0.76	0.78	–	0.88	1.00	0.93	–
Prediabetic	0.43	0.50	0.46	–	0.00	0.00	0.00	–
Overall	–	–	–	0.63	–	–	–	0.50
#3 Male, Balanced, RF, 33 features								
Normoglycemic	0.76	0.67	0.71	–	0.88	1.00	0.93	–
Prediabetic	0.31	0.42	0.36	–	0.00	0.00	0.00	–
Overall	–	–	–	0.545	–	–	–	0.50
#4 Female, Unbalanced, BMI included, LDA, 26 features								
Normoglycemic	0.78	0.74	0.76	–	0.92	0.78	0.84	–
Prediabetic	0.38	0.43	0.40	–	0.00	0.00	0.00	–
Overall	–	–	–	0.585	–	–	–	0.39
#5 Female, Unbalanced, DT, 23 features								
Normoglycemic	0.63	0.63	0.63	–	0.93	1.00	0.97	–
Prediabetic	0.00	0.00	0.00	–	0.00	0.00	0.00	–
Overall	–	–	–	0.315	–	–	–	0.50
#6 Female, Balanced, XGBoost, 24 features								
Normoglycemic	0.79	0.79	0.79	–	0.93	0.47	0.62	–
Prediabetic	0.43	0.43	0.43	–	0.06	0.50	0.11	–
Overall	–	–	–	0.61	–	–	–	0.485

Across configurations, discrimination ranged from AUC 0.65 to 0.81. In males: BNB 0.65 (0.56–0.73), XGBoost 0.66 (0.58–0.75), RF 0.68 (0.58–0.78). In females: LDA 0.68 (0.55–0.81), DT 0.69 (0.59–0.80), and XGBoost 0.81 (0.69–0.92). Calibration (Brier score) ranged 0.18–0.26, with the lowest (best) value for Female–Balanced–XGBoost (0.18). Full operating characteristics (BAcc, Sens, Spec, F1) with AUC (95% CI) and Brier are provided in **Table 2**.

Evaluation on the holdout subset of the India dataset revealed differences in model generalization across sexes and experimental configurations (**Table 1**). For female participants, the best-performing model was the same as for the cross-validation, XGBoost classifier trained on the balanced dataset, using 24 features, which achieved a balanced accuracy of 0.61. In contrast, for male participants, the top-performing model on the holdout set was the XGBoost classifier trained on the unbalanced dataset without BMI, also using 24 features, which outperformed the best from the cross-validation, the random forest model developed using the balanced dataset with balanced accuracies of 0.63 and 0.545, respectively.

To further evaluate generalizability, the top-performing models for each sex were tested on an independent external dataset collected in Canada (**Table 1**). Overall performance was substantially lower across all models when compared to the India holdout set. While most models maintained relatively strong performance in identifying normoglycemic individuals, sensitivity for prediabetes dropped sharply; in some configurations, no prediabetic participants were correctly identified. Importantly, the Canadian cohort contained very few prediabetic participants when stratified by sex, yielding unstable class-wise estimates with high variance; accordingly, the apparent collapse in prediabetic detection should be interpreted as sample-size–limited and exploratory rather than as evidence of no underlying signal. Differences in cohort composition and context (e.g., younger age distribution, linguistic/acoustic environment, and measurement/ascertainment differences) may further contribute to reduced cross-cohort generalization, so these results should be interpreted cautiously.

## 4 Discussion

This work extends prior research on voice-based detection of cardiometabolic disease by investigating whether early-stage metabolic dysfunction associated with prediabetes produces detectable changes in speech acoustics. Previous studies demonstrated that individuals with type 2 diabetes mellitus or hypertension exhibit measurable differences in vocal parameters such as pitch, jitter, and spectral complexity, achieving strong predictive performance when models were trained on these features (13, 14). In the current study, we apply a similar framework to a prediabetic population, a group in which physiological changes are expected to be more subtle. As anticipated, model accuracy was lower than in our earlier work, particularly in external validation, which may reflect the subtler physiological changes in prediabetes relative to more advanced disease.

Different experimental configurations, varying by dataset balancing and inclusion of BMI, produced varying model performances depending on the evaluation method. During development, LOSO-CV was used to evaluate configurations and select feature subsets, identifying the best-performing configurations for male and female participants within the training set. However, when these models were evaluated on an independent holdout test set, some alternative configurations that were not selected as best during LOSO-CV demonstrated superior generalization performance. This divergence highlights the well-known risk that cross-validation may favor models that perform optimally on internal folds but fail to generalize as effectively to unseen data. We also note that the holdout set was small, likely inflating the variance in balanced accuracy and class-wise metrics, therefore we interpret the holdout comparisons as indicative rather than definitive.

As a result, the final model selection must weigh both cross-validation accuracy and external validation performance. In this study, we observed that certain configurations (unbalanced dataset with BMI as an additional feature) generalized better on the holdout test set, even if they were not top-performing in LOSO-CV. Notably, external validation used an independent South Asian cohort in Canada, so ethnicity was held constant, while geography, recording context, and demographics differed. This underscores the importance of validating model performance on independent data and suggests that generalization, rather than cross-validation performance alone, should guide final model selection for clinical or real-world deployment.

The higher sensitivity observed in females may arise from biological and methodological factors. Physiological and acoustic differences between male and female voices motivated our sex-specific modeling in previous research and may make subtle metabolic effects more detectable in female voices. At the same time, dataset composition likely contributed as the female balanced set was smaller because there were fewer prediabetic females than males in the source cohort, which can alter feature selection and decision thresholds. Finally, the optimal configuration differed by sex on the holdout set (balanced for females vs. unbalanced for males), indicating that the balance–variance trade-off may interact with sex-specific vocal characteristics. Future work should examine whether sex-specific calibration, thresholding, or feature sets improve parity across sexes.

The comparison between cross-validation performance and holdout testing revealed important discrepancies in model behavior. While LOSO-CV was used during development to select the most effective models for each experimental configuration, testing on an independent holdout subset from the same India dataset identified different best-performing models. For females, the model trained on the balanced dataset without BMI achieved the highest balanced accuracy, whereas for males, a model trained on the unbalanced dataset performed better. This divergence illustrates a known limitation of cross-validation, where model selection based solely on internal validation may not always reflect the true generalization potential. These findings underscore the importance of validating model performance on unseen data, even within the same population, to avoid overfitting to cross-validation folds.

However, generalization performance further declined when these models were tested on an external dataset collected in Canada. Despite using the same modeling framework and feature set, performance dropped substantially across all metrics, particularly in detecting prediabetic individuals. Most models maintained high precision and recall for normoglycemic subjects but exhibited near-zero precision and recall for the prediabetic class. This suggests that the vocal features associated with prediabetes may not generalize well across different populations. The Canadian cohort differed meaningfully from the Indian cohort in age distribution, geographic location, and possibly linguistic, environmental, or cultural factors as each of which could influence vocal characteristics. Cross-cohort comparisons should be interpreted with caution because anthropometrics were measured in India but partially self-reported in Canada. These findings suggest that future development of voice-based predictive models may require more diverse and representative training data or the development of population-specific models that account for local demographic and environmental variability. These findings are also consistent with a true prediabetes-related vocal effect that is below the detection threshold of our current features and modeling, in addition to environmental and demographic influences.

One key limitation of this study is the potential for overfitting during model development due to the relatively small number of participants. Although a large number of voice samples were collected, feature values were averaged across all recordings per individual to produce a stable, subject-level representation. Although averaging reduces intra-subject variability, it may also attenuate potentially informative temporal dynamics (e.g. day-to-day fluctuations), which were not the target of this status classification. The number of usable recordings per participant varied widely within and between cohorts due to different completion rates, protocol differences between the India and Canada studies, and quality control which potentially introduced uneven data quality and reduced the effective information per subject. This approach, especially with sex-specific models, significantly reduced the number of training examples, increasing the risk of overfitting, particularly in a high-dimensional feature space. While feature selection and regularization were applied to mitigate this, the limited effective sample size likely constrained the model's ability to generalize. In addition, we did not perform hyperparameter optimization to minimize selection-induced bias and overfitting in this small-N setting, all classifiers were evaluated under standard default configurations with identical preprocessing and stratified cross-validation. This choice provides a fair, reproducible baseline across model families but may understate the peak performance of specific algorithms.

Another important limitation concerns the demographic differences between the India and Canada datasets, particularly in age and BMI ranges. The Canadian cohort included a much lower age and BMI range compared to the Indian cohort, which may have introduced confounding effects not captured during training. Age is known to influence vocal characteristics, and differences in geographic, cultural, or linguistic factors may have further contributed to the poor generalization observed on the external dataset. These findings highlight the need for training data that are more demographically diverse and reflective of real-world populations, or the development of population-specific models, to enhance robustness and applicability across varied settings.

One promising future direction is to evaluate whether predictive models trained to detect overt T2DM can generalize or transfer to prediabetic populations. While the current work was constrained to participants without type 2 diabetes, several of the acoustic markers found to be predictive in prior studies (e.g., pitch variability, intensity, shimmer) appear to shift subtly in the same direction among prediabetic individuals. This raises the possibility that a refined model trained on a gradient of disease severity or employing transfer learning approaches could improve early-stage detection. In parallel, increasing dataset diversity, particularly in age and dialect, and exploring semi-supervised training techniques may enhance generalizability across populations.

### 4.1 Conclusion

This study provides preliminary evidence that voice-based acoustic features may hold potential as digital biomarkers for prediabetes, warranting further validation in larger and more diverse cohorts. By evaluating multiple experimental configurations across sex-specific models and incorporating factors such as dataset balancing and BMI, we identified model configurations with moderate internal performance capable of distinguishing prediabetic from normoglycemic individuals within a local cohort. However, performance varied depending on the evaluation method, with models selected through cross-validation not always generalizing best to independent test sets. Moreover, when applied to an external population, model performance declined substantially, particularly in identifying prediabetic individuals, underscoring the challenges of cross-population generalization. These findings suggest that future voice-based predictive tools for metabolic health will require larger, more diverse datasets, as well as consideration of demographic and regional variability. Further work involving larger, more diverse datasets and hyperparameter tuning is needed to improve model performance and generalizability before voice-based screening can be considered for real-world application in prediabetes.

## Data Availability

The raw data supporting the conclusions of this article will be made available by the authors, without undue reservation upon request.
